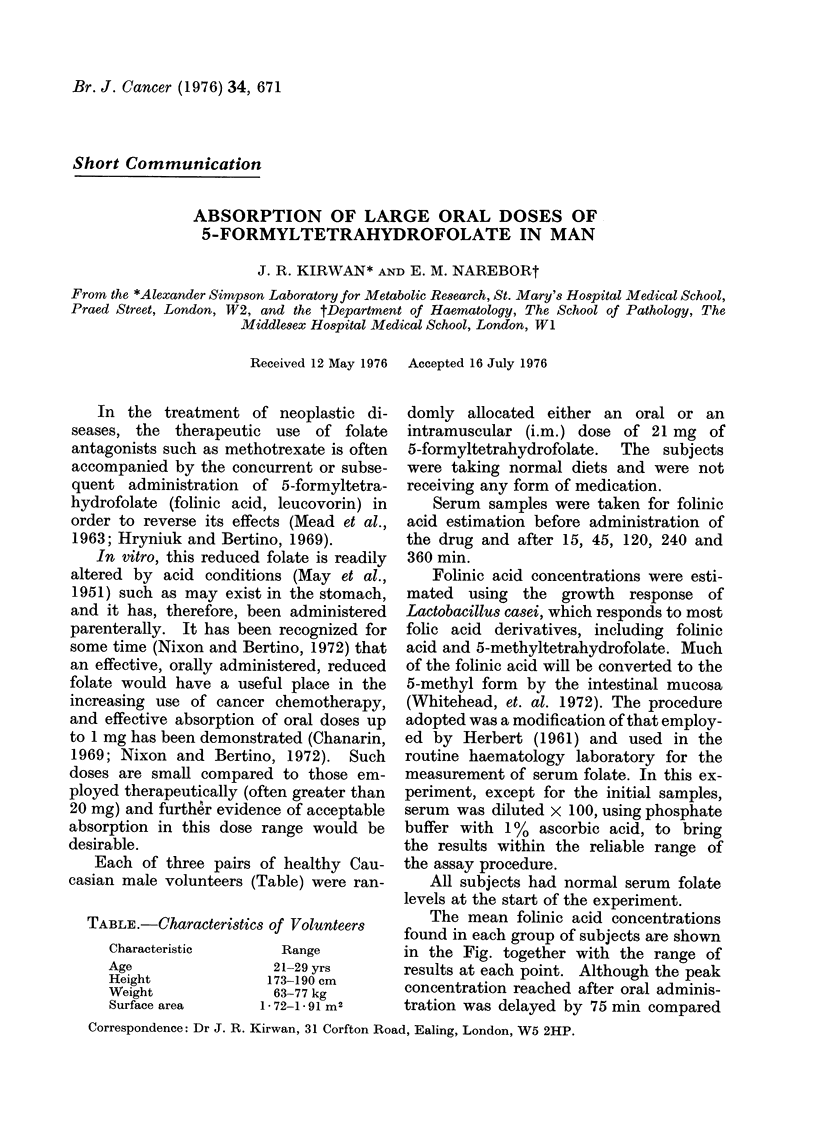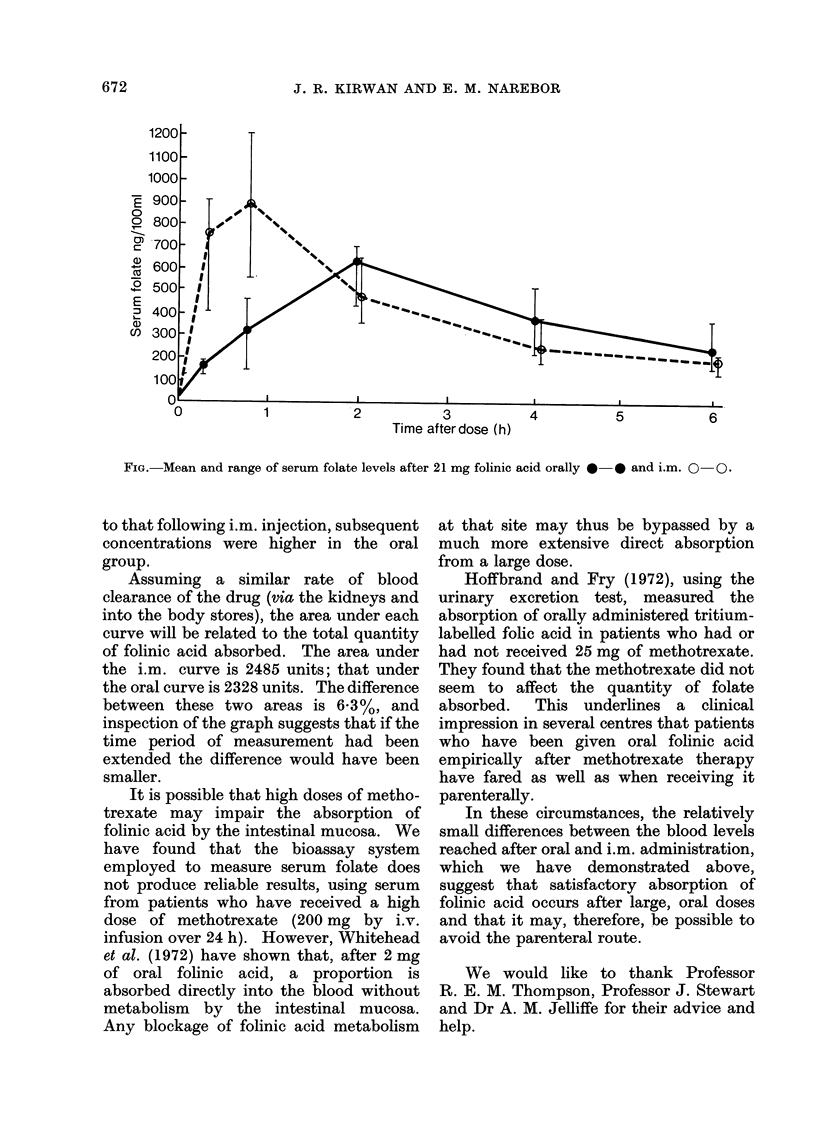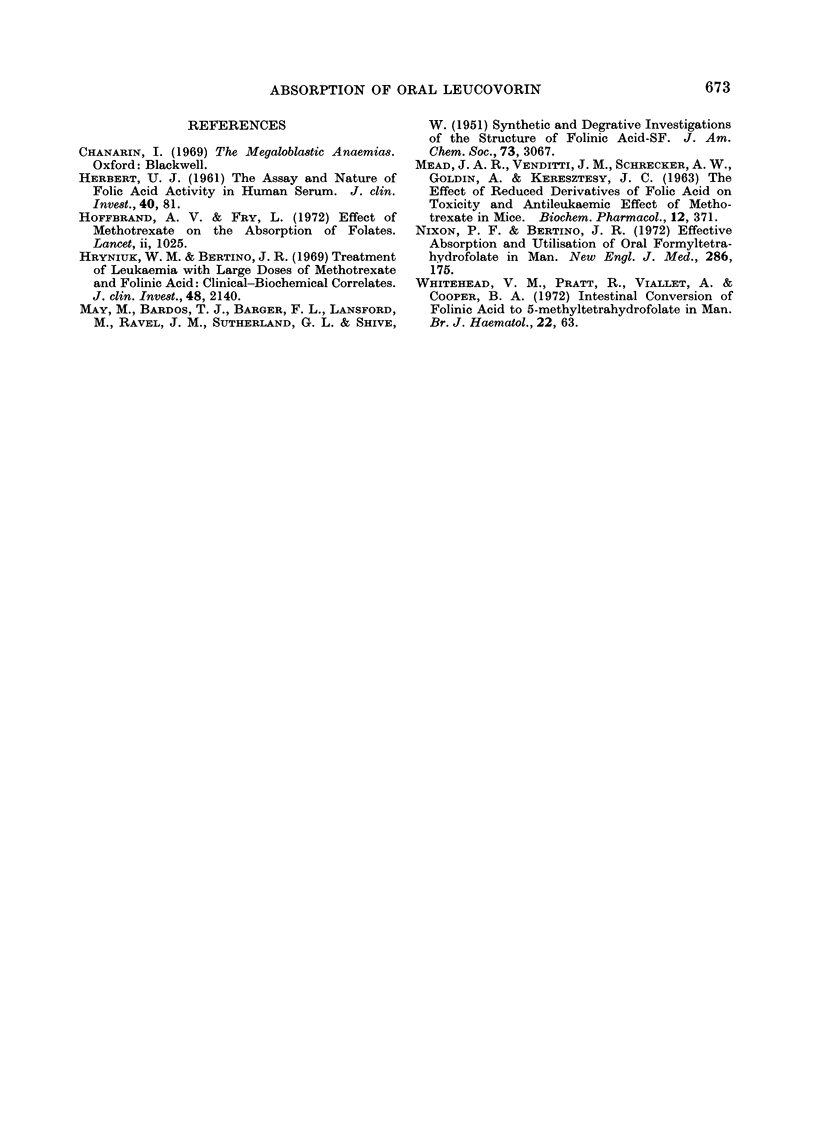# Absorption of large oral doses of 5-formyltetrahydrofolate in man.

**DOI:** 10.1038/bjc.1976.231

**Published:** 1976-12

**Authors:** J. R. Kirwan, E. M. Narebor


*Br. J. Cancer* (1976) **34**, 671

### *Short Communication*

### ABSORPTION OF LARGE ORAL DOSES OF 5-FORMYLTETRAHYDROFOLATE IN MAN

J. R. KIRWAN* AND E. M. NAREBOR†

*From the *Alexander Simpson Laboratory for Metabolic Research, St. Mary's Hospital Medical School, Praed Street, London, W2, and the †Department of Haematology, The School of Pathology, The Middlesex Hospital Medical School, London, W1*

Received 12 May 1976 Accepted 16 July 1976

In the treatment of neoplastic diseases, the therapeutic use of folate antagonists such as methotrexate is often accompanied by the concurrent or subsequent administration of 5-formyltetrahydrofolate (folinic acid, leucovorin) in order to reverse its effects (Mead *et al.*, 1963; Hryniuk and Bertino, 1969).

*In vitro*, this reduced folate is readily altered by acid conditions (May *et al.*, 1951) such as may exist in the stomach, and it has, therefore, been administered parenterally. It has been recognized for some time (Nixon and Bertino, 1972) that an effective, orally administered, reduced folate would have a useful place in the increasing use of cancer chemotherapy, and effective absorption of oral doses up to 1 mg has been demonstrated (Chanarin, 1969; Nixon and Bertino, 1972). Such doses are small compared to those employed therapeutically (often greater than 20 mg) and further evidence of acceptable absorption in this dose range would be desirable.

Each of three pairs of healthy Caucasian male volunteers (Table) were randomly allocated either an oral or an intramuscular (i.m.) dose of 21 mg of 5-formyltetrahydrofolate. The subjects were taking normal diets and were not receiving any form of medication.

TABLE.—*Characteristics of Volunteers*

| Characteristic | Range |
|---|---|
| Age | 21–29 yrs |
| Height | 173–190 cm |
| Weight | 63–77 kg |
| Surface area | 1·72–1·91 m² |

Serum samples were taken for folinic acid estimation before administration of the drug and after 15, 45, 120, 240 and 360 min.

Folinic acid concentrations were estimated using the growth response of *Lactobacillus casei*, which responds to most folic acid derivatives, including folinic acid and 5-methyltetrahydrofolate. Much of the folinic acid will be converted to the 5-methyl form by the intestinal mucosa (Whitehead, *et. al.* 1972). The procedure adopted was a modification of that employed by Herbert (1961) and used in the routine haematology laboratory for the measurement of serum folate. In this experiment, except for the initial samples, serum was diluted × 100, using phosphate buffer with 1% ascorbic acid, to bring the results within the reliable range of the assay procedure.

All subjects had normal serum folate levels at the start of the experiment.

The mean folinic acid concentrations found in each group of subjects are shown in the Fig. together with the range of results at each point. Although the peak concentration reached after oral administration was delayed by 75 min compared

Correspondence: Dr J. R. Kirwan, 31 Corfton Road, Ealing, London, W5 2HP.

FIG.—Mean and range of serum folate levels after 21 mg folinic acid orally ●—● and i.m. ○—○.

to that following i.m. injection, subsequent concentrations were higher in the oral group.

Assuming a similar rate of blood clearance of the drug (*via* the kidneys and into the body stores), the area under each curve will be related to the total quantity of folinic acid absorbed. The area under the i.m. curve is 2485 units; that under the oral curve is 2328 units. The difference between these two areas is 6·3%, and inspection of the graph suggests that if the time period of measurement had been extended the difference would have been smaller.

It is possible that high doses of methotrexate may impair the absorption of folinic acid by the intestinal mucosa. We have found that the bioassay system employed to measure serum folate does not produce reliable results, using serum from patients who have received a high dose of methotrexate (200 mg by i.v. infusion over 24 h). However, Whitehead *et al.* (1972) have shown that, after 2 mg of oral folinic acid, a proportion is absorbed directly into the blood without metabolism by the intestinal mucosa. Any blockage of folinic acid metabolism at that site may thus be bypassed by a much more extensive direct absorption from a large dose.

Hoffbrand and Fry (1972), using the urinary excretion test, measured the absorption of orally administered tritium-labelled folic acid in patients who had or had not received 25 mg of methotrexate. They found that the methotrexate did not seem to affect the quantity of folate absorbed. This underlines a clinical impression in several centres that patients who have been given oral folinic acid empirically after methotrexate therapy have fared as well as when receiving it parenterally.

In these circumstances, the relatively small differences between the blood levels reached after oral and i.m. administration, which we have demonstrated above, suggest that satisfactory absorption of folinic acid occurs after large, oral doses and that it may, therefore, be possible to avoid the parenteral route.

We would like to thank Professor R. E. M. Thompson, Professor J. Stewart and Dr A. M. Jelliffe for their advice and help.

### REFERENCES

CHANARIN, I. (1969) *The Megaloblastic Anaemias.* Oxford: Blackwell.

HERBERT, U. J. (1961) The Assay and Nature of Folic Acid Activity in Human Serum. *J. clin. Invest.*, **40**, 81.

HOFFBRAND, A. V. & FRY, L. (1972) Effect of Methotrexate on the Absorption of Folates. *Lancet*, ii, 1025.

HRYNIUK, W. M. & BERTINO, J. R. (1969) Treatment of Leukaemia with Large Doses of Methotrexate and Folinic Acid: Clinical–Biochemical Correlates. *J. clin. Invest.*, **48**, 2140.

MAY, M., BARDOS, T. J., BARGER, F. L., LANSFORD, M., RAVEL, J. M., SUTHERLAND, G. L. & SHIVE, W. (1951) Synthetic and Degrative Investigations of the Structure of Folinic Acid-SF. *J. Am. Chem. Soc.*, **73**, 3067.

MEAD, J. A. R., VENDITTI, J. M., SCHRECKER, A. W., GOLDIN, A. & KERESZTESY, J. C. (1963) The Effect of Reduced Derivatives of Folic Acid on Toxicity and Antileukaemic Effect of Methotrexate in Mice. *Biochem. Pharmacol.*, **12**, 371.

NIXON, P. F. & BERTINO, J. R. (1972) Effective Absorption and Utilisation of Oral Formyltetrahydrofolate in Man. *New Engl. J. Med.*, **286**, 175.

WHITEHEAD, V. M., PRATT, R., VIALLET, A. & COOPER, B. A. (1972) Intestinal Conversion of Folinic Acid to 5-methyltetrahydrofolate in Man. *Br. J. Haematol.*, **22**, 63.